# Digital Quantification of Intratumoral CD8+ T-Cells Predicts Relapse and Unfavorable Outcome in Uveal Melanoma

**DOI:** 10.3390/cancers14235959

**Published:** 2022-12-02

**Authors:** Ozge Hurdogan, Francesco De Logu, Francesca Galli, Samuray Tuncer, Filippo Ugolini, Sara Simi, Francesca Portelli, Romina Nassini, Daniela Massi, Nesimi Buyukbabani

**Affiliations:** 1 Department of Pathology, Istanbul Faculty of Medicine, Istanbul University, Istanbul 34093, Turkey; 2 Department of Health Sciences, Section of Clinical Pharmacology and Oncology, University of Florence, 50100 Florence, Italy; 3 Methodology for Clinical Research Laboratory, Oncology Department, Istituto di Ricerche Farmacologiche Mario Negri IRCCS, 20156 Milan, Italy; 4 Department of Ophthalmology, Istanbul Faculty of Medicine, Istanbul University, Istanbul 34093, Turkey; 5 Department of Health Sciences, Section of Pathological Anatomy, University of Florence, 50100 Florence, Italy; 6 Klinisk Patologi och Cancerdiagnostik, Karolinska Universitetssjukhuset, 17176 Solna, Sweden

**Keywords:** uveal melanoma, tumor microenvironment, prognosis, immunohistochemistry

## Abstract

**Simple Summary:**

In this study, we used digital pathology and image analysis for the prediction of the prognosis in uveal melanoma (UM). We retrospectively evaluated a total of 404 histopathological slides of 101 patients. Immune biomarkers for CD4, CD8, CD68, and CD163 were performed and evaluated by digital image acquisition and quantitative analysis. Our results showed that a higher intratumoral CD8 positive cell density imposed a negative impact on RFS and OS. Our study also showed that older age and stage III were independent negative prognostic factors for both RFS and OS. Our results demonstrate that a specific distribution profile of CD8 may imply a higher risk of relapse and death in UMs, and further studies could elaborate specific subgroups that are amenable to various treatment regimens.

**Abstract:**

Although it is a disease that occurs mainly in the Caucasian population, uveal melanoma (UM) is the most common primary intraocular tumor in adults. Here, we used digital pathology and image analysis for the diagnosis of UM and the prediction of the prognosis. Our retrospective study included a total of 404 histopathological slides from 101 patients. A digital image acquisition and quantitative analysis of tissue immune biomarkers (CD4, CD8, CD68, CD163) were performed. A negative impact of the intratumoral CD8 positive cell density higher than 13.3 cells/mm^2^ was detected for both RFS (HR 2.08, 95% Cl 1.09 to 3.99, *p* = 0.027) and OS (HR 3.30, 95% CI 1.58 to 6.88, *p* = 0.001). Moreover, we confirmed that older age and stage III were independent negative prognostic factors for both RFS and OS. Our results suggest that a specific distribution profile of CD8 in UM might predict the risk of relapse and death, with potential implications for determining which subgroups of UMs are amenable to specific pharmacological treatment regimens.

## 1. Introduction

Uveal melanoma (UM) is the most common primary ocular malignancy in adults and a highly aggressive and biologically distinct tumor [1]. The incidence of UM is reported as being 2–8/1,000,000 in the Western world [1,2]. The large majority of UMs (>90%) arise from the choroid, followed by the ciliary body and/or iris, with or without choroidal involvement [3]. Tumor size, ciliary body involvement, old age, inflammatory infiltration, extra-scleral invasion, and epithelioid cell type have been reported as unfavorable prognostic factors [3,4,5,6]. Eye-confined disease is treated either by vision-saving therapies such as brachytherapy and proton beam radiotherapy or by enucleation [7,8]. Despite recent improvements in tumor local control, UMs have a high propensity to metastasize [2,9,10]. Approximately 50% of patients develop metastases within 10 years from the diagnosis, and the survival rates of patients with metastatic disease remain poor [11].

The eye is considered an immune privileged site, where both innate and adaptive immunity are regulated with peculiar immunosuppressive mechanisms, different compared to those of other anatomical sites [12,13,14]. Indeed, the ocular microenvironment is characterized by a strong representation of immune suppressive molecules that make the eye immunologically unique [12,13,14].

It is known that the immune microenvironment is an important prognostic factor in cancer, and immune cell infiltration is often associated with a favorable prognosis [15]. In the context of UM, inflammatory cell infiltration has been described within and at the periphery of the tumor, especially when epithelioid cells are predominant [7]. High densities of tumor-associated macrophages (TAMs) and tumor-infiltrating lymphocytes (TILs) in primary UM have been correlated with a higher metastasis risk and worse prognosis [16,17,18]. Previous studies have shown that most TAMs in UMs are M2-type cells expressing CD68+ and CD163+, and survival was significantly reduced among patients with a high M2 macrophage and T cell density [19,20,21,22]. The response to immunotherapy with pembrolizumab was correlated to high numbers of CD8+, PD-1+, and PDL-1+ cells, as well as an increase in CD8+ cell infiltration during treatment [23,24].

In this study, we aimed to assess the spatial distribution and prognostic impact of inflammatory cell infiltration in UM tissue samples. Digital whole slide imaging scan and image analysis software were used to resolve the quantitative density of CD4+ and CD8+ lymphocytes as well as CD68+ and CD163+ macrophages and to establish the possible correlation between inflammatory cell infiltration and the clinical outcome.

## 2. Material and Methods

### 2.1. Case Selection, Clinical and Pathological Data Collection

The study cohort included formalin-fixed paraffin-embedded (FFPE) tissues from 101 UM patients diagnosed between 2004 and 2019 and retrospectively retrieved from the archives of Istanbul University, Istanbul Faculty of Medicine, Department of Pathology. Patients’ clinical data including follow-up information were obtained from the Department of Ophthalmology’s records. The use of FFPE sections of human samples was approved by the Local Ethics Committee (2020/1654), according to the Helsinki Declaration, and informed consent was obtained.

### 2.2. Immunohistochemistry

FFPE tissue sections, 3 μm in thickness, were stained with hematoxylin and eosin and centrally reviewed to assess tissue quality control. Samples’ processing was performed with a Ventana Discovery XT immunostainer (Ventana Medical Systems, Tucson, AZ, USA). The sections were deparaffinized in EZ prep (950-102; Ventana), and antigen retrieval was achieved by incubation with cell-conditioning solution 1 (950-124; Ventana) (pH 8.2) for 32 min at 100 °C. Sections were incubated with the following primary antibodies: anti-CD4 (rabbit monoclonal, clone SP35, ready to use, Ventana Medical System, Tucson, AZ, USA), anti-CD68 (mouse monoclonal, clone PG-M1, ready to use, Diagnostic BioSystem, Pleasanton, CA, USA), anti-CD8 (rabbit monoclonal, clone SP57, ready to use, Ventana Medical System, Tucson, AZ, USA), and anti-CD163 (mouse monoclonal, clone MRQ-26, ready to use, Ventana Medical System, Tucson, AZ, USA). For all antibodies, the signal was developed with the UltraMap Red anti-mouse or anti-rabbit Detection Kit (Ventana Medical Systems, Tucson, AZ, USA). Sections were counterstained with hematoxylin.

The intratumoral inflammatory cells positive for CD4, CD8, CD68, CD163 were semi-quantitatively assessed under light microscopy by one of the authors (OH). Specifically, score 0 implied no interstitial inflammatory cells, score 1: scattered inflammatory cells, score 2: conspicuous inflammatory cell infiltration under low magnification, and score 3: diffuse/clustering infiltration.

### 2.3. Digital Image Analysis

Stained tissue sections were digitally scanned at a ×400 magnification with the Aperio AT2 platform (Leica Biosystems, Wetzlar, Germany) into whole slide digital images (WSI). Individual SVS format files were imported into HALO digital imaging analysis software (Indica Labs, Albuquerque, NM, USA). Two expert pathologists (DM and NB) drew the image annotations of the whole surface of UM. Using the module Indica Labs-Multiplex IHC v3.1.4, we performed TILs and TAMs detection based on cytonuclear features such as stain intensity, size, and roundness for CD4, CD8, CD68, and CD163 positive cells. The software automatically excludes tissue gaps from analysis, and the settings were set to include the full range of staining intensities (from weak to strong). In pigmented UM samples, an exclusion mask was set for the melanin pigment, in order to discriminate the brownish melanin from a positive red signal, eliminating the risk of an overestimation of the staining. Whole tissue slides from 101 cases underwent image analysis for the 4 markers previously specified. HALO counted the intratumoral CD4+, CD8+, CD68+, and CD163+ cells. Of the 404 IHC sections, slide detachments were observed in 7 during tissue staining. Thus, 397/404 (98.2%) of the samples remained available for evaluation. Data were collected as the cellular density and as the number of positive cells divided by the tissue annotation area (mm^2^).

### 2.4. Statistical Analyses

The study aimed to evaluate the prognostic value of intratumoral immune cell density on relapse-free survival (RFS) and overall survival (OS) in UM patients. RFS was defined as the time between diagnosis and disease relapse or death from any cause. OS was defined as the time between diagnosis and death from any cause. Patients who had not relapsed/died or died were censored at the date of the last follow-up visit. Each immune cell biomarker was evaluated as a continuous variable and categorized as low or high according to its median.

Continuous variables were described using the mean and standard deviation (SD), the median with the first and third quartiles (Q1–Q3; interquartile range, IQR), and minimum and maximum values, whereas categorical variables were described using frequencies and percentages. The association between the immune cell biomarkers was assessed by means of the Spearman correlation index. RFS and OS were evaluated using the univariable and multivariable Cox proportional hazard models.

Multivariable models were adjusted for the age, sex, and stage at the diagnosis. Results of the analyses were expressed as hazard ratios (HRs) and 95% confidence intervals (95% CIs). The median RFS and OS were estimated with the Kaplan-Meier (KM) method. Statistical significance was set at *p* < 0.05 for a bilateral test. Analysis was carried out using the SAS (Statistical Analysis System, SAS Institute, Cary, NC, USA, Version 9.4) software.

## 3. Results

### 3.1. Clinical Features

Among the 101 analyzed samples, information on the diagnosis date, relapse, or survival was not available for 29 patients. Therefore, 72 patients (37 males and 35 females) with a median age of 61 years (IQR 46.5 to 72.5 years) and diagnosed with UM between April 2004 and December 2013 were included in the study. Only one (1.4%) patient had exenteration, while the remaining 71 (98.6%) patients underwent enucleation. The median interval between the diagnosis and the surgery was 0.7 months (IQR 0.3 to 1.4 months). The majority of the tumors (n = 64, 88.9%) originated from choroid, followed by ciliary body (n = 5, 6.9%), iris (n = 2, 2.8%), and both choroid and ciliary body (n = 1, 1.4%). Four (5.6%) patients received neoadjuvant treatment (chemo/radiotherapy). Presenting symptoms were commonly described as a loss of vision, sudden floaters, and photopsia.

### 3.2. Histopathology

Tumor samples had a median largest diameter equal to 17.0 mm (IQR 13.5 to 20.0 mm). The tumor cell type was mixed in 38 (52.8%) cases, spindle in 26 (36.1%) cases, and epithelioid in eight (11.1%) cases. A weak, moderate, and marked pigmentation was observed in 24 (33.3%), 30 (41.7%), and 18 (25.0%) samples, respectively. The tumors were classified at diagnosis as stage IIA in 2 (2.8%) cases, stage IIB in 32 (44.4%) cases, stage IIIA in 22 (30.6%) cases, stage IIIB in 12 (16.7%) cases, and stage IIIC in 4 (5.6%) cases. The 8th edition of the American Joint Committee on Cancer (AJCC) TNM (Tumor, Node, and Metastasis) Staging System has been used for staging. The clinical and pathological characteristics of the study cases are listed in the Supplementary Table (Table S1).

### 3.3. Immunohistochemistry and Digital Image Analysis

**Figure 1:**
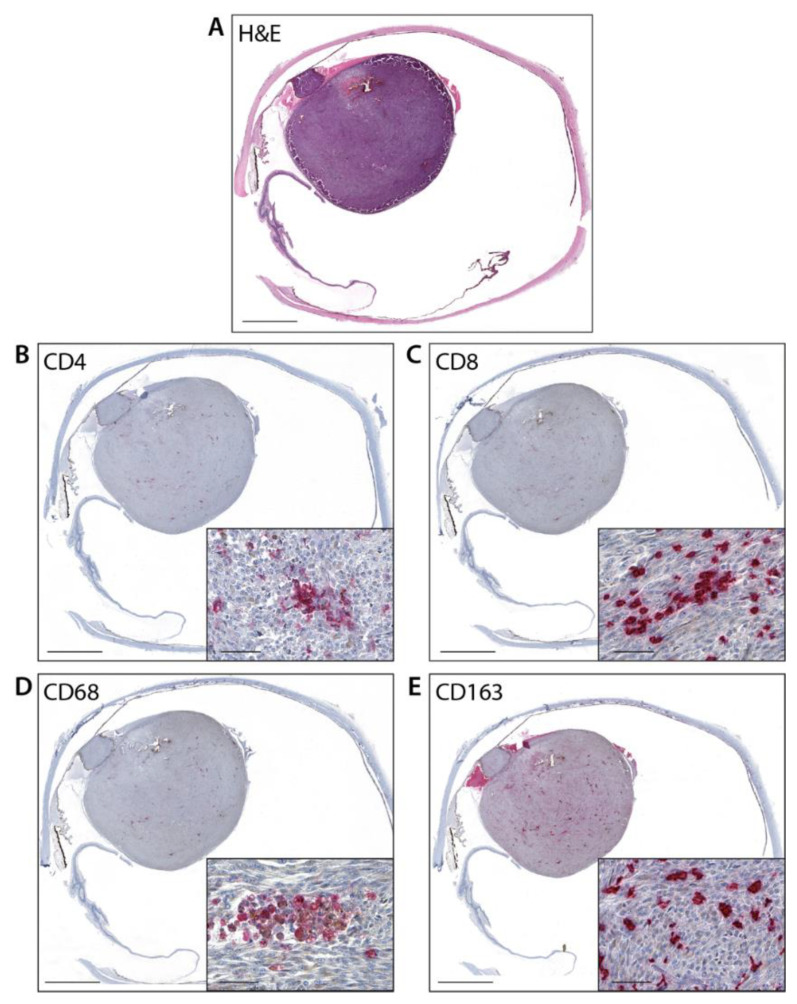
Representative image of UM tissue. (**A**): Hematoxylin & Eosin stain (Magnification ×10; scale bar 200 μm); (**B**): Intratumoral CD4+ expression (Magnification ×10, inset ×200; scale bar 200 μm, 50 μm, respectively); (**C**): Intratumoral CD8+ expression (Magnification ×10, inset ×200; scale bar 200 μm, 50 μm, respectively); (**D**): Intratumoral CD68+ expression (Magnification ×10, inset ×200; scale bar 200 μm, 50 μm, respectively); (**E**): Intratumoral CD163+ expression (Magnification ×10, inset ×200; scale bar 200 μm, 50 μm, respectively).

The semiquantitative intratumoral inflammatory cell scores detected on immunohistochemical slides as well as the cell density (cell number/mm^2^) value for each antibody (CD4, CD8, CD68, CD163) are listed in Table 1, and representative images of staining are shown in Figure 1 (A-E). A moderate positive correlation was observed between the cell densities of CD4+ and CD8+ (rho 0.57) and between CD8+ and CD163+ (rho 0.56). A lower correlation was detected between CD4+ and CD163+ (rho 0.41) and between CD68+ and CD163+ (rho 0.31).

### 3.4. Follow-Up and Survival Information

The median follow-up time was 9.0 years (IQR 7.3 to 10.0). Twenty-eight (38.9%) patients showed relapse. Metastases were mostly detected in the liver (19 patients, 67.9%). Other rarer metastatic sites were the brain, bone, adrenal, and lungs. Thirty-five (48.6%) patients died. Out of the 25 patients with a known death cause, only three (12.0%) deaths were non-disease-related. Overall, 41 (56.9%) patients relapsed or died. The median RFS was 6.9 years (3.2—third quartile not reached), and the median OS was 8.7 years (4.6—third quartile not reached).

**Figure 2:**
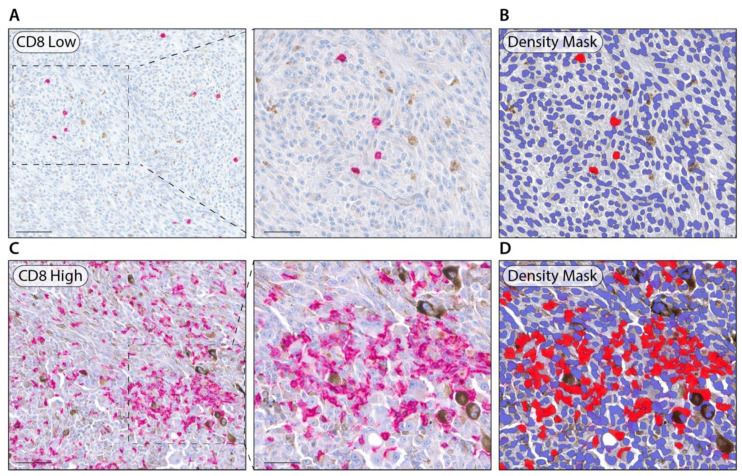
(**A**): Representative UM tissues showing intratumoral low CD8+ expression (Magnification ×200, ×400; scale bar 50 μm, 20 μm, respectively); (**B**): Representative HALO density recognition mask of low CD8+ expression; (**C**): Representative UM tissues showing intratumoral high CD8+ expression (Magnification ×200, ×400; scale bar 50 μm, 20 μm, respectively); (**D**): Representative density recognition mask of high CD8+ expression.

**Figure 3:**
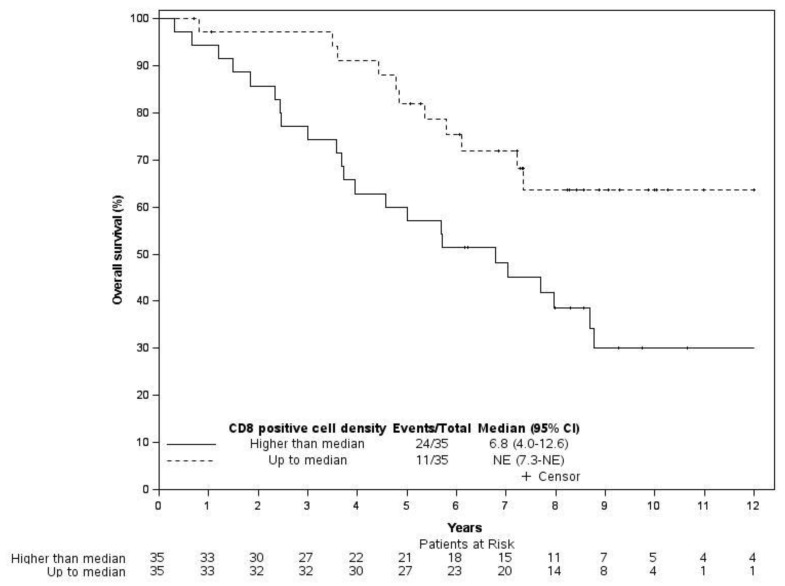
Overall survival graphs for CD8+ cell density.

The results of the univariable analyses on RFS and OS are summarized in the Supplementary Table (Table S2). No statistically significant effects of the inflammatory cell densities on RFS were detected, while an intratumoral CD8+ cell density higher than 13.3 cells/mm^2^ showed a negative impact on OS (HR 2.48, 95% Cl 1.21 to 5.09, *p* = 0.013). Representative UM tissues showing intratumoral low/high CD8+ expressions are shown in Figure 2. The KM curves of OS according to the CD8 positive cell density are depicted in Figure 3. Moreover, an older age significantly shortened both RFS (HR [1 year increase] 1.03, 95% Cl 1.01 to 1.06, *p* = 0.003) and OS (HR [1 year increase] 1.05, 95% Cl 1.02 to 1.07, *p* < 0.001). Moreover, patients diagnosed at stage III had a statistically significantly worse RFS (HR 2.47, 95% Cl 1.29 to 4.73, *p* = 0.007) and OS (HR 2.84, 95% Cl 1.36 to 5.91, *p* = 0.005) compared to stage II patients.

Table 2 and Table 3 provide the results of multivariable analyses on RFS and OS. A negative impact of the intratumoral CD8 positive cell density higher than 13.3 cells/mm^2^ was detected for both RFS (HR 2.08, 95% CI 1.09 to 3.99, *p* = 0.027) and OS (HR 3.30, 95% Cl 1.58 to 6.88, *p* = 0.001). Lastly, we confirmed that older age and stage III were independent negative prognostic factors for both RFS and OS.

## 4. Conclusions

Here, we showed that intratumoral CD8+ lymphocytes are associated with worse OS in uni- and multivariable analyses as well as poorer RFS in multivariable analyses, as opposed to what happens in many solid tumors, in which a high presence of TILs is an indicator of a good prognosis [25]. Despite literature data on inflammatory cell infiltration and UM microenvironment focusing mostly on the role of macrophages, there is evidence about the correlation of lymphocyte infiltration and survival in UMs [26]. There is a potential relationship, showing a worse prognosis, between the inflammatory infiltrate and UMs within the Class II subgroup based on the gene expression profile [27]. Other evidence reinforced the relationship between immune cell infiltration and prognosis, with the exception of some genetically distinct subgroups [28]. Recently, a bioinformatic analysis and CD8+ gene signature confirmed the prognostic role of CD8 in UM [17,18]. In addition, a recent study performing single cell analyses of the tumor and the microenvironment in primary and metastatic UMs outlined genomic complexity, as well as revealing a different subset of CD8+ T cells expressing checkpoint marker LAG3 but not PD1 or CTLA4 [29].

These studies suggested that the high immunosuppressive tumor microenvironment (TME) present in UMs caused a failure in the activation of CD8+ cells, which could not express their strong antitumoral function [17]. Unfortunately, in this context, the host’s immune system could inadvertently promote tumor growth [30].

M1-type macrophages possess anti-tumoral properties, while M2 macrophages promote tumor growth through immunosuppression and are related to a worse prognosis in several cancer types [31,32]. Studies have shown the relation of CD68+ macrophages with a worse prognosis [2] and the fact that reduced CD68+ and CD163+ cell counts improve survival [21]. The increase in macrophages was linked to a larger tumor diameter and epithelioid cell type [2], as well as ciliary body involvement [21]. Gezgin et al. proposed an association between the inflammatory phenotype and genetic evolution [20]. The presence of monosomy of chromosome 3 increases the M2 macrophage and T cell infiltration, worsens survival, and is linked to metastasis and death [20,21,33]. In our study, cases with higher CD68+ and CD163+ cell counts showed worse RFS and OS. However, the statistical analysis did not show a significant difference.

Literature data shows that macrophage activation precedes T cell activation [20]. Macrophages can activate both the T cells and tumor progression by stimulating angiogenesis and by creating an immunosuppressive microenvironment [21]. A study based on genetic T cell quantification revealed that all UMs with a dismal prognosis showed T cell influx and that T cell-related gene expression signatures were mostly derived from macrophages. This phenomenon demonstrates the relation between these two cell types [34].

Almost half of our cases succumbed to disease, and more than half showed relapse and metastasis. The liver was affected in the majority of metastases, which also corresponds to several other studies in the literature [35,36,37]. The median OS was 104 months, which is shorter than in most reported series [38,39]. Another study reported that all patients with metastatic disease died [40]. Based on the TNM classification by AJCC, most of our cases were diagnosed at Stage IIB, followed by Stage IIIA, which may explain the relatively lower survival rates. Moreover, in accordance with previously published papers [41,42], we confirmed that older age and stage III were independent prognostic factors in our cohort of patients.

We may infer that higher CD8+ cells are correlated with an immune evasive tumor microenvironment, which explains the poor prognosis. The density of CD8+ cell infiltration needs to be genetically validated for further interpretation, which is a limitation of our study.

In conclusion, our study has analyzed the immune contexture using a software-based image analysis of a large monocentric cohort of UM patients with long-term follow-up, and we found that a high intratumoral CD8+ cell score is a negative prognostic indicator for RFS and OS. Further elaboration of UM’s biological characteristics may help researchers to determine which subgroups of UMs are amenable to specific pharmacological treatment regimens. Novel treatments may ensue from the accrual of further data on the microenvironment of UM.

## Figures and Tables

**Table 1:** Score and automated density of immunohistochemistry and Digital Image Analysis in uveal melanomas.

	Overall N = 72
**CD4+ intratumoral score**	
0	37 (51.4)
1+	31 (43.1)
2+	4 (5.6)
**CD4+ density (cells/mm^2^)** *	
Mean (SD)	126.2 (199.8)
Median (Q1–Q3)	39.4 (8.6–122.2)
Min–Max	0.0–760.1
**CD8+ intratumoral score**	
0	17 (23.6)
1+	39 (54.2)
2+	13 (18.1)
3+	3 (4.2)
**CD8+ density (cells/mm^2^)** †	
Mean (SD)	113.3 (213.0)
Median (Q1–Q3)	13.3 (3.0–124.7)
Min–Max	0.0–939.0
**CD68+ intratumoral score** *	
0	12 (16.9)
1+	34 (47.9)
2+	22 (31.0)
3+	3 (4.2)
**CD68+ density (cells/mm^2^)** *	
Mean (SD)	99.3 (167.4)
Median (Q1–Q3)	46.1 (4.1–106.8)
Min–Max	0.0–846.4
**CD163+ intratumoral score**	
0	2 (2.8)
1+	10 (13.9)
2+	32 (44.4)
3+	28 (38.9)
**CD163+ density (cells/mm^2^)** ††	
Mean (SD)	337.5 (295.2)
Median (Q1–Q3)	260.6 (96.4–524.6)
Min–Max	0.0–1188.0

* Total n = 71; † Total n = 70; †† Total n = 69.

**Table 2:** Multivariable analysis of relapse free survival (RFS).

	Multivariable Model Including CD4+		Multivariable Model Including CD8+		Multivariable Model Including CD68+		Multivariable Model Including CD163+	
	HR (95% CI)	*p*-Value	HR (95% CI)	*p*-Value	HR (95% CI)	*p*-Value	HR (95% CI)	*p*-Value
**CD4+ density (cells/mm^2^)** (>39.4 vs. ≤39.4)	0.96 (0.51–1.79)	0.891						
**CD8+ density (cells/mm^2^)** (>13.3 vs. ≤13.3)			2.08 (1.09–3.99)	**0.027**				
**CD68+ density (cells/mm^2^)** (>46.1 vs. ≤46.1)					1.12 (0.58–2.17)	0.745		
**CD163+ density (cells/mm^2^)** (>260.6 vs. ≤260.6)							1.73 (0.91–3.27)	0.094
**Age** (1 year increase)	1.03 (1.01–1.06)	**0.008**	1.04 (1.01–1.06)	**0.003**	1.03 (1.01–1.06)	**0.007**	1.03 (1.01–1.05)	**0.015**
**Sex** (Male vs. Female)	1.17 (0.63–2.19)	0.623	1.21 (0.64–2.28)	0.564	1.15 (0.62–2.16)	0.656	1.07 (0.57–2.00)	0.832
**Stage at diagnosis** (III vs. II)	2.43 (1.25–4.71)	**0.009**	2.86 (1.44–5.68)	**0.003**	2.37 (1.21–4.65)	**0.012**	2.88 (1.44–5.76)	**0.003**

Bold number only for significant *p* values.

**Table 3:** Multivariable analysis of overall survival (OS).

	Multivariable Model Including CD4+		Multivariable Model Including CD8+		Multivariable Model Including CD68+		Multivariable Model Including CD163+	
	HR (95% CI)	*p*-Value	HR (95% CI)	*p*-Value	HR (95% CI)	*p*-Value	HR (95% CI)	*p*-Value
**CD4+ density (cells/mm^2^)** (>39.4 vs. ≤39.4)	1.12 (0.57–2.21)	0.745						
**CD8+ density (cells/mm^2^)** (>13.3 vs. ≤13.3)			3.30 (1.58–6.88)	**0.001** *				
**CD68+ density (cells/mm^2^)** (>46.1 vs. ≤46.1)					1.26 (0.61–2.61)	0.528		
**CD163+ density (cells/mm^2^)** (>260.6 vs. ≤260.6)							1.93 (0.96–3.90)	0.067
**Age** (1 year increase)	1.05 (1.02–1.08)	**0.001**	1.05 (1.02–1.08)	**<0.001**	1.05 (1.02–1.08)	**0.001**	1.05 (1.02–1.08)	**0.001**
**Sex** (Male vs. Female)	1.34 (0.67–2.67)	0.412	1.58 (0.79–3.17)	0.196	1.32 (0.66–2.64)	0.436	1.26 (0.63–2.52)	0.521
**Stage at diagnosis** (III vs. II)	2.68 (1.27–5.64)	**0.010**	2.89 (1.36–6.12)	**0.006**	2.59 (1.21–5.52)	**0.014**	3.22 (1.49–6.98)	**0.003**

*: significant *p* value

## Data Availability

The data presented in this study are available upon request.

## Supplementary Materials

The following supporting information can be downloaded at: https://www.mdpi.com/article/10.3390/cancers14235959/s1, Table S1. Clinicopathological Features of the Study Cohort. Table S2. Univariable Analysis on Relapse Free Survival (RFS) and Overall Survival (OS).
