# Supplementary material for: Digital Quantification of Intratumoral CD8+ T-Cells Predicts Relapse and Unfavorable Outcome in Uveal Melanoma
Source: Cancers (Basel). 2022 Dec 2;14(23):5959. doi: 10.3390/cancers14235959 (PMC9740732; doi:10.3390/cancers14235959)
Supplement: Supplementary file 1 [file cancers-14-05959-s001.zip › cancers-1932914-supplementary.pdf]

Table S1. Clinicopathological Features of The Study Cohort.

Case No	Age (years)	Gender	Tumor localization	Type of Surgery	Largest tumor diameter (mm)	TNM (VIII ed.)	Stage at diagnosis
1	46	Male	Choroid (ciliary body invasion)	Left Enucleation	16	T4dN0M0	IIIC
2	81	Female	Choroid (ciliary body invasion)	Right Enucleation	23	T4bN0M0	IIIB
3	73	Male	Choroid	Right Enucleation	20	T4aN0M0	IIIA
4	55	Male	Ciliary body	Right Enucleation	12,3	T3bN0M0	IIIA
5	55	Male	Choroid	Left Enucleation	20	T4dN0M0	IIIC
6	47	Female	Choroid	Left Enucleation	15	T3aN0M0	IIB
7	62	Female	Choroid (ciliary body invasion)	Left Enucleation	20	T4bN0M0	IIIB
8	44	Male	Choroid	Left Enucleation	16	T4aN0M0	IIIA
9	71	Male	Choroid	Left Enucleation	12	T3aN0M0	IIB
10	73	Male	Ciliary body (choroid invasion)	Left Enucleation	20	T4bN0M0	IIIB
11	78	Male	Choroid (ciliary body invasion)	Left Enucleation	23	T4bN0M0	IIIB
12	48	Male	Iris (ciliary body invasion)	Right Enucleation	1,5	T3aN0M0	IIB
13	74	Male	Choroid	Right Enucleation	18	T2aN0M0	IIA
14	27	Female	Choroid	Right Enucleation	14	T3aN0M0	IIB
15	20	Male	Choroid	Right Enucleation	21	T4aN0M0	IIIA
16	61	Male	Choroid (ciliary body invasion)	Right Enucleation	18	T4bN0M0	IIIB
17	61	Male	Choroid	Left Enucleation	10	T3aN0M0	IIB
18	64	Male	Choroid	Left Enucleation	17	T3aN0M0	IIB
19	77	Female	Choroid (ciliary body invasion)	Left Enucleation	15	T3bN0M0	IIIA
20	69	Male	Ciliary body (choroid invasion)	Left Enucleation	16	T4bN0M0	IIIB
21	69	Female	Choroid (ciliary body invasion)	Left Enucleation	14	T4bN0M0	IIIB
22	63	Female	Choroid	Right Enucleation	20	T4aN0M0	IIIA
23	55	Male	Choroid	Right Enucleation	24	T4aN0M0	IIIA
24	40	Male	Choroid	Right Enucleation	12	T3aN0M0	IIB
25	64	Male	Ciliary body	Right Enucleation	10	T2bN0M0	IIB
26*	69	Female	Choroid	Right Enucleation	16	T3aN0M0	IIB
27	47	Male	Choroid	Left Enucleation	17	T3aN0M0	IIB
28	56	Male	Choroid	Right Enucleation	19	T4bN0M0	IIIB
29	50	Female	Choroid	Left Enucleation	13	T3aN0M0	IIB
30	30	Female	Choroid	Left Enucleation	18	T3aN0M0	IIB
31	76	Male	Choroid	Right Enucleation	21	T3aN0M0	IIB
32	46	Female	Choroid+Ciliary body	Right Enucleation	16	T4bN0M0	IIIB
33	36	Male	Choroid	Left Enucleation	9	T3aN0M0	IIB
34*	50	Male	Choroid	Right Enucleation	14	T4aN0M0	IIIA
35	53	Male	Choroid	Right Enucleation	13	T3aN0M0	IIB
36*	69	Male	Choroid	Left Enucleation	18	T3aN0M0	IIB

37	68	Male	Choroid	Right Enucleation	18	T4cN0M0	IIIB
38*	80	Male	Choroid	Right Enucleation	19	T4aN0M0	IIIA
39	46	Female	Choroid	Left Enucleation	12	T3aN0M0	IIB
40	47	Female	Choroid	Right Enucleation	15	T4aN0M0	IIIA
41	76	Female	Choroid	Left Enucleation	19	T3aN0M0	IIB
42	62	Female	Choroid	Right Enucleation	14	T3aN0M0	IIB
43*	66	Female	Choroid	Right Enucleation	10	T3aN0M0	IIB
44*	56	Male	Choroid	Left Enucleation	12	T2aN0M0	IIA
45	41	Female	Choroid	Left Enucleation	11	T3aN0M0	IIB
46*	46	Female	Choroid	? Enucleation	10	T2aN0M0	IIA
47	74	Female	Choroid (ciliary body invasion)	? Enucleation	18	T3bN0M0	IIIA
48	74	Male	Choroid	Left Exenteration	60	T4eN0M0	IIIC
49	42	Male	Choroid	Left Enucleation	17	T3aN0M0	IIB
50	60	Male	Choroid	Left Enucleation	21	T4bN0M0	IIIB
51	45	Male	Choroid	Left Enucleation	17	T3aN0M0	IIB
52	71	Male	Choroid	Right Enucleation	12	T3aN0M0	IIB
53	72	Male	Choroid	Right Enucleation	10	T3aN0M0	IIB
54	68	Male	Choroid	Right Enucleation	17	T3aN0M0	IIB
55*	66	Female	Choroid	Left Enucleation	16	T3aN0M0	IIB
56*	89	Female	Choroid	Right Enucleation	13	T3aN0M0	IIB
57	55	Female	Choroid	Left Enucleation	15	T3aN0M0	IIB
58	65	Male	Choroid	Right Enucleation	15	T3aN0M0	IIB
59	78	Female	Choroid	Right Enucleation	19	T4aN0M0	IIIA
60*	74	Female	Choroid	? Enucleation	18	T3aN0M0	IIB
61*	54	Male	Choroid	Left Enucleation	20	T3aN0M0	IIB
62	74	Female	Choroid	Left Enucleation	25	T4aN0M0	IIIA
63*	81	Female	Choroid	Left Enucleation	12	T3aN0M0	IIB
64*	68	Female	Choroid (ciliary body invasion)	Left Enucleation	20	T4dN0M0	IIIC
65	95	Female	Choroid	Left Enucleation	12	T3aN0M0	IIB
66	71	Female	Iris	Right Enucleation	9	T3aN0M0	IIB
67	70	Female	Choroid	Right Enucleation	15	T3aN0M0	IIB
68	49	Female	Choroid	Left Enucleation	17	T3aN0M0	IIB
69	51	Male	Choroid	Right Enucleation	14	T2aN0M0	IIA
70	48	Male	Choroid	Right Enucleation	17	T4aN0M0	IIIA
71	43	Female	Choroid	Left Enucleation	22	T4aN0M0	IIIA
72*	73	Female	Choroid	Left Enucleation	14	T2aN0M0	IIA
73*	66	Male	Choroid	Right Enucleation	17	T3aN0M0	IIB
74*	53	Male	Choroid	Left Enucleation	17	T3aN0M0	IIB
75	65	Female	Choroid	Right Enucleation	22	T4aN0M0	IIIA
76	80	Female	Choroid	Left Enucleation	9,5	T3aN0M0	IIB
77*	70	Male	Choroid	Left Enucleation	7	T1aN0M0	I
78	78	Female	Choroid	Right Enucleation	19	T4aN0M0	IIIA
79*	67	Female	Choroid	Left Enucleation	15	T3aN0M0	IIB
80	34	Female	Choroid	Left Enucleation	23	T4aN0M0	IIIA
81	65	Male	Choroid	Right Enucleation	18	T4aN0M0	IIIA
82*	86	Female	Choroid	Left Enucleation	15	T3aN0M0	IIB
83	76	Female	Choroid	Right Enucleation	20	T4aN0M0	IIIA
84	28	Female	Choroid	Right Enucleation	12	T3aN0M0	IIB

85*	27	Male	Ciliary body (iris invasion)	Left Enucleation	10	T1aN0M0	I
86*	62	Female	Choroid	Right Enucleation	12	T2aN0M0	IIA
87*	59	Female	Choroid	Right Enucleation	14	T3aN0M0	IIB
88*	73	Male	Choroid	Right Enucleation	15	T3aN0M0	IIB
89	90	Female	Choroid	Left Enucleation	18	T4aN0M0	IIIA
90	61	Female	Choroid	Left Enucleation	11,25	T4aN0M0	IIIA
91*	62	Female	Choroid	Left Enucleation	14	T3aN0M0	IIB
92	39	Male	Choroid	Left Enucleation	22	T4eN0M0	IIIC
93*	78	Female	Choroid+Ciliary body	Left Enucleation	16	T3bN0M0	IIIA
94*	60	Male	Choroid	Right Enucleation	17	T3aN0M0	IIB
95*	36	Female	Choroid	Left Enucleation	22	T4aN0M0	IIIA
96	82	Female	Choroid	Right Enucleation	20	T4cN0M0	IIIB
97*	58	Male	Choroid	Left Enucleation	20	T4eNxMx	IIIC
98	61	Male	Choroid	Left Enucleation	18	T3bN0M0	IIIA
99	31	Female	Ciliary body (choroid invasion)	Right Enucleation	18	T3bN0M0	IIIA
100	39	Female	Choroid	Right Enucleation	18	T3aN0M0	IIB
101*	61	Male	Choroid (ciliary body invasion)	Right Enucleation	24	T4eN0M0	IIIC

*Those cases are excluded from the survival analyses for being lost to follow-up.

Table S2. Univariable analysis on relapse free survival (RFS) and overall survival (OS).

	**Relapse free survival**	**Overall survival**
	**HR (95% CI), p-value**	**HR (95% CI), p-value**
**CD4+ score** (1+/2+ vs 0)	1.20 (0.65-2.22), 0.561	1.47 (0.75-2.88), 0.260
**CD4+ density** (10 cell/mm^2 increase)	1.00 (0.99-1.02), 0.699	1.01 (0.99-1.02), 0.450
**CD4+ density (cell/mm^2)** (>39.4 vs ≤ 39.4)	0.89 (0.48-1.65), 0.721	1.05 (0.54-2.04), 0.885
**CD8+ score** (ref. 0)	0.323	0.142
1+	1.55 (0.66-3.62), 0.311	1.26 (0.50-3.21), 0.621
2+ 3+	2.07 (0.80-5.36), 0.133	2.40 (0.88-6.52), 0.086
**CD8+ density** (10 cell/mm^2 increase)	1.00 (0.99-1.02), 0.642	1.01 (0.99-1.02), 0.251
**CD8+ density (cell/mm^2)** (>13.3 vs ≤ 13.3)	1.61 (0.85-3.04), 0.144	2.48 (1.21-5.09), **0.013**
**CD68+ score** (ref. 0)	0.981	0.704
1+	1.09 (0.46-2.60), 0.846	1.10 (0.40-3.00), 0.856
2+ 3+	1.06 (0.43-2.62), 0.896	1.42 (0.51-3.96), 0.498
**CD68+ density** (10 cell/mm^2 increase)	0.99 (0.97-1.02), 0.621	1.00 (0.98-1.02), 0.944
**CD68+ density (cell/mm^2)** (>46.1 vs ≤ 46.1)	0.92 (0.50-1.71), 0.796	0.88 (0.45-1.71), 0.706
**CD163+ score** (ref. 0/1)	0.539	0.252
2+	1.45 (0.57-3.65), 0.435	2.73 (0.80-9.32), 0.109
3+	1.70 (0.67-4.35), 0.267	2.72 (0.79-9.44), 0.114
**CD163+ density** (10 cell/mm^2 increase)	1.00 (0.99-1.01), 0.411	1.00 (0.99-1.01), 0.494
**CD163+ density (cell/mm^2)** (>260.6 vs ≤ 260.6)	1.45 (0.78-2.68), 0.240	1.57 (0.80-3.07), 0.189
**Age** (1 years increase)	1.03 (1.01-1.06), **0.003**	1.05 (1.02-1.07), **<0.001**
**Sex** (Male vs Female)	1.11 (0.60-2.05), 0.745	1.15 (0.59-2.24), 0.679
**Stage at diagnosis** (III vs II)	2.47 (1.29-4.73), **0.007**	2.84 (1.36-5.91), **0.005**

Note: Significant p-values in bold.
